# Community-Acquired Pneumonia due to *Streptococcus pneumoniae*: When to Consider Coinfection with Active Pulmonary Tuberculosis

**DOI:** 10.1155/2019/4618413

**Published:** 2019-09-05

**Authors:** Ruslan Garcia

**Affiliations:** NewYork-Presbyterian Hospital/Weill Cornell Medical Center, New York, NY, USA

## Abstract

Community-acquired pneumonia (CAP) is an important cause of hospitalizations in adults. In the United States, *Streptococcus pneumoniae* is the most frequently identified bacterial pathogen responsible for CAP. Other etiologic pathogens of CAP vary based on the geographic region. *Mycobacterium tuberculosis* is an uncommon cause of CAP in the United States, while it is a principal cause in many African and Asian countries. Coinfection with *Streptococcus pneumoniae* and *Mycobacterium tuberculosis* is rare and has only been reported in the setting of underlying HIV infection in areas of high tuberculosis prevalence. Here, we report a case of CAP in the absence of HIV, where *Streptococcus pneumoniae* was identified on admission and delay in diagnosis of concomitant active pulmonary tuberculosis led to inappropriate isolation. In addition to a high index of suspicion, epidemiologic and radiographic findings can be helpful to recognize tuberculosis as a cause of CAP even when other pathogens have already been identified.

## 1. Introduction

*Streptococcus pneumoniae* (i.e., pneumococcus) is the most frequently identified bacterial pathogen responsible for community-acquired pneumonia (CAP) in the United Sates (US), albeit with a decreasing incidence given the use of pneumococcal vaccines [1]. The Centers for Disease Control and Prevention (CDC) report that pneumococcal pneumonia leads to approximately 400,000 hospitalizations per year in the US, with greater morbidity and mortality in children and in the elderly [2].

Other etiologic agents of CAP can vary based on the geographic region. In the US, *Mycobacterium tuberculosis* (MTB) is an uncommon cause of CAP, whereas it remains one of the main etiologic agents in many other countries, especially in Africa and Asia [3, 4]. MTB is responsible for significant morbidity and mortality, as it is the leading infectious cause of death and the tenth overall cause of death worldwide [5].

CAP caused by coinfection of *Streptococcus pneumoniae* and *Mycobacterium tuberculosis* has been previously reported only in areas of high MTB prevalence and in the setting of active HIV infection [6]. Here, we report a case of CAP caused by coinfection with pneumococcus and active pulmonary tuberculosis (TB) in the absence of HIV infection.

## 2. Case Presentation

An 87-year-old Chinese man with a history of diabetes and ischemic cardiomyopathy presented with one week of productive cough of green sputum and dyspnea. He denied any fevers, chills, night sweats, or weight loss. He did not have any recent travel or exposure to sick contacts. His last travel to China or outside the United States was 40 years ago. On presentation, he was afebrile and hemodynamically stable with a blood pressure of 123/74, a heart rate of 71, and an oxygen saturation of 97% on room air. Physical examination was otherwise notable for nonlabored breathing at rest with scattered bilateral crackles throughout both lung fields.

**Figure 1:**
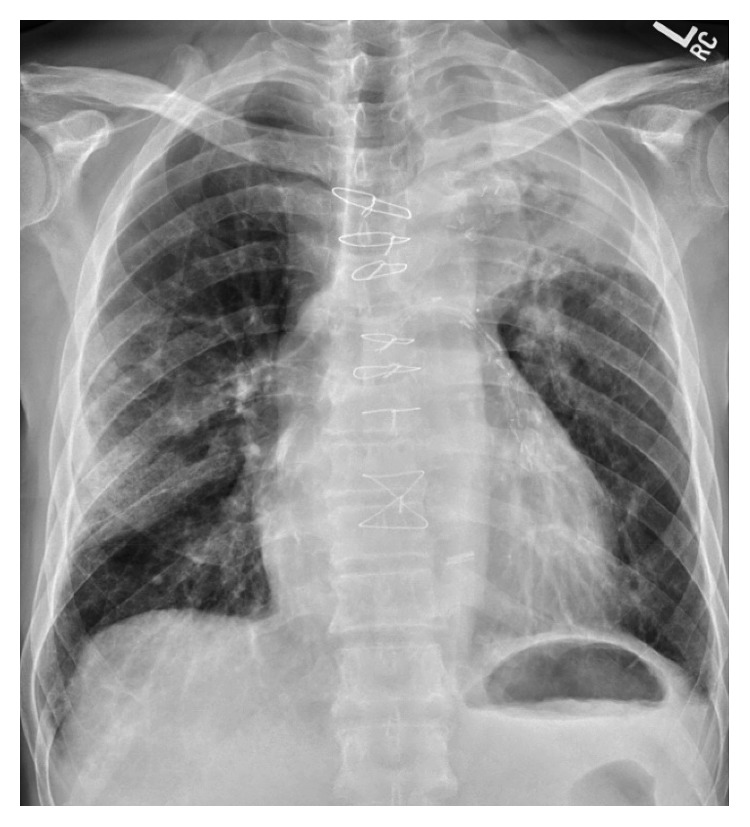
Chest radiograph on admission: multifocal pneumonia.

**Figure 2:**
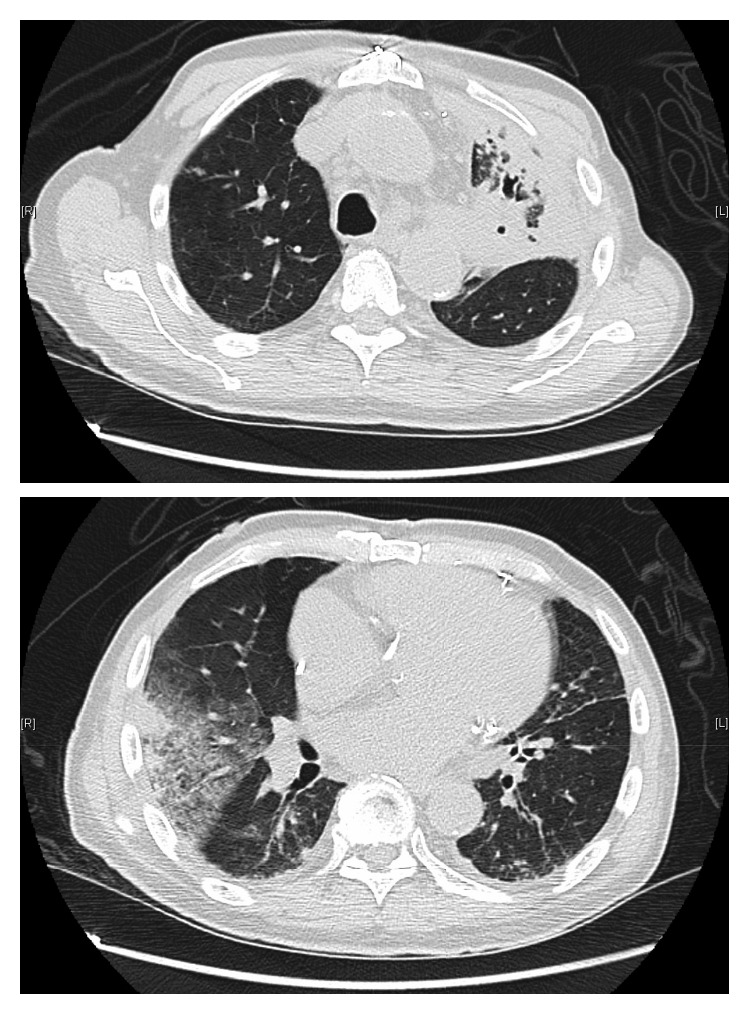
CT chest scan on admission: bronchiectasis and consolidation in the left upper lobe in addition to peripheral consolidation in the right upper lobe with adjacent ground-glass opacification.

Infectious labs were initially significant for a white blood cell count of 10.5 × 10^3^/*μ*L, a procalcitonin level of 0.54 ng/mL, and a positive *Streptococcus pneumoniae* urine antigen. Other labs including respiratory viral panel, *Legionella* urine antigen, and blood cultures were negative. A chest radiograph on admission was consistent with a multifocal pneumonia (see Figure 1). A computed tomography (CT) scan of the chest without contrast confirmed multilobar opacities, including the left apical and upper lobe consolidations with bronchiectasis, as well as a right upper lobe consolidation with ground-glass opacification (see Figure 2). A small calcified granuloma was also noted in the right lower lobe. Testing for TB with an interferon-gamma release assay was considered but not performed given that an etiologic agent was already identified.

The patient was started on ceftriaxone 1 gram every 24 hours for empiric coverage of *Streptococcus pneumoniae*. On hospital day 4, he became increasingly somnolent and hypoxic, requiring intermittent nasal canula despite four doses of ceftriaxone. Repeat chest X-ray revealed worsening bilateral infiltrates. Ceftriaxone was increased to 2 gram every 24 hours, and vancomycin was added to cover for possible beta-lactam resistance.

**Figure 3:**
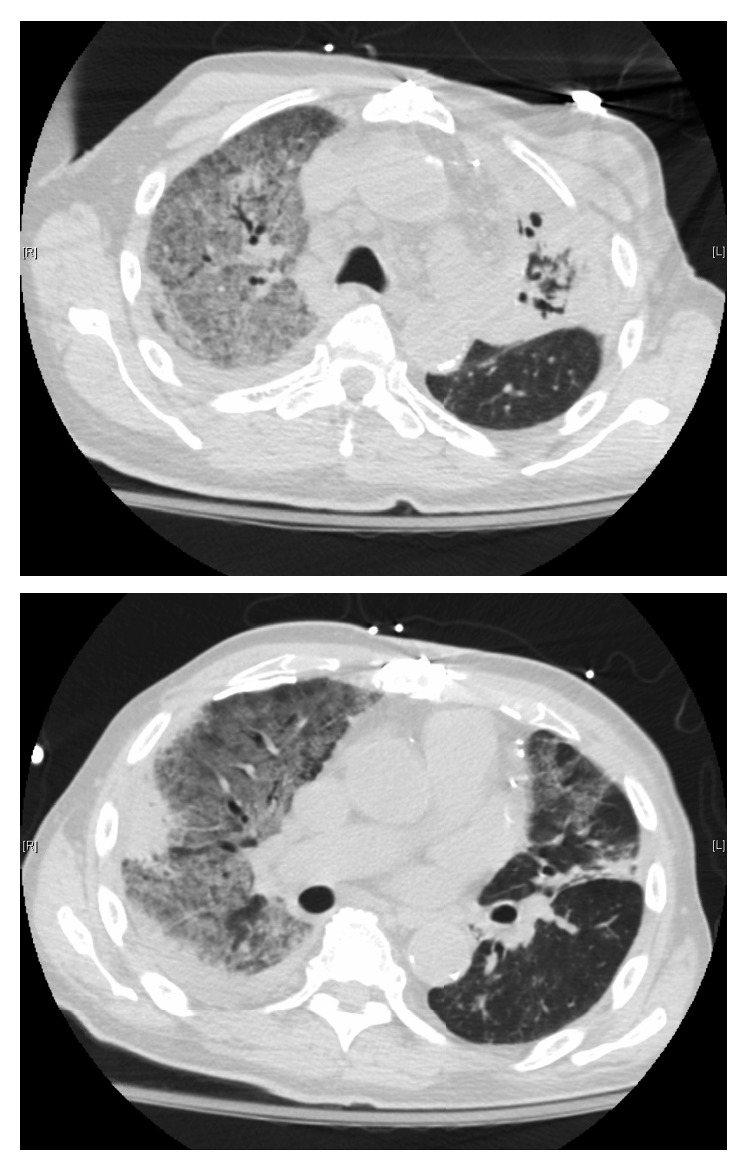
CT chest scan on hospital day 6: persistent consolidation in the left upper lobe with bronchiectasis and in the right upper lobe again noted; new scattered areas of ground glass are seen in both lungs, particularly affecting the right upper lobe.

On hospital day 6, he developed rigors, tachypnea, and labored breathing. Arterial blood gas analysis revealed a pH, carbon dioxide (CO_2_), and lactate of 7.36, 18, and 7, respectively. At this point, heart failure with volume overload and acute respiratory distress syndrome were considered. Aggressive diuresis was started, and antibiotics were escalated to piperacillin-tazobactam, azithromycin, and vancomycin. He was also placed on bilevel positive airway pressure for work of breathing. Repeat CT scan of the chest revealed persistent bilateral consolidations with diffuse ground-glass opacities and small pleural effusions (see Figure 3). On hospital day 7, respiratory status deteriorated despite noninvasive positive pressure ventilation, and emergent intubation with bronchoalveolar lavage (BAL) was performed. He died shortly after intubation. Subsequently, BAL studies returned positive for acid-fast bacilli which speciated as *Mycobacterium tuberculosis*.

## 3. Discussion

Identifying the causative pathogen for patients hospitalized with community-acquired pneumonia (CAP) is important to promote the use of targeted antibiotic therapy and to provide appropriate public health guidance. However, even with an extensive inpatient workup, identification of a causative pathogen is elusive in more than 50% of cases [7].

Given that *Streptococcus pneumoniae* is the most common bacterial cause of CAP in our population, the pneumococcal urine antigen test (PUAT) is a routine part of the inpatient evaluation of pneumonia at this institution. The positive likelihood ratio for the PUAT has been reported to be 15–20 [8].

Pneumococcal pneumonia classically presents within days as a febrile illness with productive cough (e.g., rusty sputum), dyspnea, and pleuritic chest pain [9]. Radiographic findings are variable and commonly include unilateral or bilateral lobar consolidations (including apical and upper lung zones), ground-glass attenuation, and pleural effusions [10].

Pulmonary TB can exist as an active or latent disease. In the US, most cases of active TB occur in the context of reactivation of latent disease (i.e., postprimary TB) during episodes of systemic illness [11]. Active TB classically presents after weeks to months of symptoms and is characterized by cough (e.g., hemoptysis), fevers, night sweats, and weight loss. Typical radiographic findings in reactivation TB include apical and upper lung zone consolidations as well as cavitary lesions [12].

Given that this particular patient presented with respiratory symptoms of one-week duration, bilateral consolidations with ground glass, and a positive PUAT, it was postulated that the entire presentation was attributable to pneumococcal pneumonia. In retrospect, there are factors in this case that should have increased the pretest probability of coinfection with active pulmonary TB.

In the US, foreign-born persons are 15 times more likely to have TB, and this individual was born in China where TB is endemic [13]. The classic lobar distributions of reactivation TB were present, including apical and upper lobe consolidations. Additionally, while no prior radiographs were available for comparison, there was evidence to suggest chronic infection given the findings of bronchiectasis and a small calcified granuloma. Lastly, older age and presence of diabetes, a relatively immunocompromising condition, both increase the risk for the reactivation of latent TB [14]. Taken together, these findings should have increased suspicion for active TB so as to trigger appropriate airborne isolation and testing.

In terms of testing for TB, an interferon-gamma release assay (IGRA) was considered to aid in ruling out active disease. However, this test was disregarded because it fares poorly in the setting of active infection likely secondary to an impaired immune system, with Sester et al. reporting a pooled sensitivity and specificity of 80% and 79%, respectively [15]. Appropriate diagnostic tests for active pulmonary TB include nucleic acid amplification (NAA) as well as sputum smear and culture [16].

## 4. Conclusion/Recommendations

This is the first reported case of CAP caused by coinfection with *Streptococcus pneumoniae* and *Mycobacterium tuberculosis* in the absence of HIV. It is likely that the pneumococcal pneumonia may have provided fertile ground for latent TB to reactivate. Alternatively, it is also possible that he had subclinical pulmonary TB with impaired lung architecture in the setting of poorly controlled diabetes, and this provided a suitable environment for aggressive pneumococcal infection. Given the rapid clinical deterioration, we also postulate that the major driver of mortality in this case was the pneumococcal pneumonia rather than the pulmonary TB, which tends to be more insidious as mentioned previously. It is unlikely that starting anti-TB therapy would have provided any benefit acutely.

The major consequence of this case is the failure of appropriate isolation, which placed patients in close proximity as well as providers at risk. To prevent this recurrence, we recommend testing all individuals who present with CAP for TB even if an alternative diagnosis is already made when (1) there is significant exposure to endemic TB areas and (2) there is a characteristic TB lesion, such as an upper lung infiltrate. Diagnosing TB in areas of low prevalence such as the US is often difficult and requires a high index of suspicion, especially when other potential etiologies are identified. The gold standard test remains sputum smear and culture, although the World Health Organization recommends nucleic acid amplification in conjunction for rapid diagnosis [16]. The IGRA should not be used if active infection is suspected.
